# Evaluation of a short version of the Experiences in Close Relationships-Revised questionnaire (ECR-RD8): results from a representative German sample

**DOI:** 10.1186/s40359-021-00637-z

**Published:** 2021-09-14

**Authors:** Johannes C. Ehrenthal, Johannes Zimmermann, Katja Brenk-Franz, Ulrike Dinger, Henning Schauenburg, Elmar Brähler, Bernhard Strauß

**Affiliations:** 1 Department of Psychology, University of Cologne, Bernhard-Feilchenfeld-Str. 11, 50969 Cologne, Germany; 2 Department of Psychology, University of Kassel, Kassel, Germany; 3 Institute of Psychosocial Medicine, Psychotherapy and Psychooncology, Jena University Hospital, Jena, Germany; 4 Department of General Internal Medicine and Psychosomatics, University Hospital Heidelberg, Heidelberg, Germany; 5 Department of Medical Psychology and Medical Sociology, University Hospital Leipzig, Leipzig, Germany; 6 Department for Psychosomatic Medicine and Psychotherapy, University Medical Center Mainz, Mainz, Germany

**Keywords:** Attachment, Assessment, Screening, ECR-R, Norm-values

## Abstract

**Background:**

Attachment insecurity is a prominent risk factor for the development and course of psychiatric and psychosomatic disorders. The Experiences in Close Relationships - Revised (ECR-R) questionnaire is a widely used self-report to assess attachment related anxiety and avoidance. However, its length has the potential to restrict its use in large, multi-instrument studies. The aim of this study was to develop and evaluate a brief version of the ECR-R, and provide norm values for the German population.

**Methods:**

A screening version of the original ECR-R was developed through principal components analysis of datasets from several previous studies. In a representative sample of 2428 randomly selected individuals from the German population, we compared fit indices of different models by means of confirmatory factor analyses (CFA). We investigated the convergent validity of the screening version in an independent convenience sample of 557 participants. Correlations between the short and the full scale were investigated in a re-analysis of the original German ECR-R evaluation sample.

**Results:**

CFA indicated a satisfactory model fit for an eight-item version (ECR-RD8). The ECR-RD8 demonstrated adequate reliability. The subscales correlated as expected with another self-report measure of attachment in an independent sample. Individuals with higher levels of attachment anxiety, but especially higher levels of attachment avoidance were significantly more likely to not be in a relationship, across all age groups. Correlations between the short and the full scale were high.

**Conclusions:**

The ECR-RD8 appears to be a reliable, valid, and economic questionnaire for assessing attachment insecurity. In addition, the reported population-based norm values will help to contextualize future research findings.

**Supplementary Information:**

The online version contains supplementary material available at 10.1186/s40359-021-00637-z.

## Background

### Attachment as a developmental risk factor

Individual differences in attachment are important, as they influence interpersonal and intrapersonal reactions to stress and strain, thereby impacting a variety of factors associated with psychiatry and health psychology [1, 2]. Attachment insecurity is a risk-factor for the development of mental disorders [3], and contributes to higher levels of psychopathology in cross-sectional analyses [1]. It is associated with several somatic and psychosomatic disease conditions [4, 5], probably via interrelated behavioral and biopsychological pathways [6], and interacts with other developmental variables in predicting psychophysiological stress-reaction [7]. In addition, there is considerable evidence for a positive impact of attachment security on psychotherapy outcome [8] and higher levels of working alliance [9–11]. Attachment styles and representations can change through psychosocial treatment [12], and the therapist’s own secure internal working models of attachment may help when treating especially challenging patients [13]. The general importance of attachment in psychiatric assessment is also acknowledged in the construct ‘affiliation and attachment’ of the NIMH Research Domain Criteria approach [14].

Key features of the attachment behavioral system are internal working models (IWM) of attachment. These are social-cognitive schemata that start to form in early childhood and are relatively stable across the lifespan [15]. The IWM integrate attachment-related experiences, and shape emotional, cognitive, and motivational patterns of attachment security, avoidance, and anxiety. Secure individuals are confident that others will be there for them in times of need, comfortable with depending on others, but they also trust their self-regulatory abilities. Avoidant individuals usually prefer to handle distress by themselves, as they expect others not to be available or competent enough to help. Anxious individuals tend to underestimate their own regulatory abilities, and seek help to the point of being dependent on others. On a psychological level, these patterns are associated with two regulatory strategies: hyperactivation as the main strategy of attachment anxiety, and deactivation as the main strategy of attachment avoidance. Both operate on levels of pre-emptive as well as subsequent regulatory defensive strategies against emotional distress associated with unmet attachment-needs [16, 17].

### Models of attachment and their assessment

The assessment of attachment patterns, styles, and representations follows two research traditions. The developmental-clinical tradition historically preferred behavioral tasks such as the Strange Situation [18], or linguistic analyses of interview-based narratives, such as the Adult Attachment Interview [19, 20]. Both have the advantage of assessing attachment dynamics and regulatory strategies while provoking the activation of the attachment system by either separation-reunification-sequences or specific questions on attachment-related topics. Similarly, in addition to three organized attachment patterns (secure, anxious, avoidant), they provide diagnostic material for a classification of disorganization of the attachment system. Attachment disorganization is related to psychopathology over and above other variants of attachment insecurity [21]. At the same time, behavior- and interview-based measures of attachment are time-consuming. One Adult Attachment Interview (AAI) may last for 40 to 120 min, and needs to be transcribed before the rating procedure can start. Reliability of each coder is established during a period of at least 18 months, following a two-week training seminar [19]. While their scientific and clinical value is undisputed, the use of these measures in most clinical settings and large studies is limited.

Another way of assessing attachment styles evolved from social psychology and research on intimate relationships [22]. Here, individuals describe themselves on self-report questionnaires regarding attachment-related experiences, expectations, and goals. Attachment questionnaires have evolved from single-item measures to complex and domain-specific instruments [22]. They share a common ground of conceptualizing attachment as a dimensional construct, often – but not exclusively – by mapping the items on the dimensions of attachment-related anxiety and avoidance [17, 23]. While the empirical associations between interview- and questionnaire-based measures of attachment are small [24], questionnaire data has successfully been applied to predict automatic reaction tendencies and behavior in clinical and non-clinical samples [25].

### The ECR-R and its German version

One of today’s most widely used attachment questionnaire is the Experiences in Close Relationships - Revised questionnaire (ECR-R) [26]. It captures attachment-related cognitions and expectations with regard to romantic relationships on two scales: Attachment anxiety and avoidance. Each scale comprises 18 items, resulting in a total length of 36 items. The ECR-R was developed through a re-analysis of a pool of 323 attachment questionnaire items from a former study. Fraley and colleagues [26] first used cluster-analytic techniques to group items with high within-group conceptual similarity, but high between-group differences. Thirty resulting clusters were then assessed by principal-axis factor analysis and rotated to fit two dimensions of attachment anxiety and avoidance. Items best representing one of these dimensions, while having low factor loadings on the respective other one, were used to create the two ECR-R scales. Their test information functions, and thus their measurement precision, were substantially better than those of an earlier instrument, the ECR [27]. The ECR-R is widely used in all areas of psychology, psychiatry, and related disciplines, and has been translated into major languages of the world.

The German version of the ECR-R, the ECR-RD, was translated in agreement with R. Chris Fraley and examined for its psychometric properties and validity by Ehrenthal and colleagues [28] in a sample of 1006 participants from a convenience sample, and 225 individuals currently in psychotherapy treatment. The ECR-RD correlated as expected with the related subscales of the Relationship Questionnaire (RQ), a brief common measure that assesses general self-view with regard to attachment following the model by Bartholomew and Horowitz [29, 30]. For example, higher scores on the ECR-RD attachment anxiety subscales were associated with higher levels of RQ subscales “preoccupied” and “fearful”, and lower levels of the RQ subscale “secure”. ECR-RD attachment avoidance was positively associated with RQ “dismissive”, RQ “fearful”, and in one of the two samples slightly with RQ “preoccupied”, and negatively with RQ “secure” [28]. In addition, both satisfaction with a current relationship as well as life satisfaction in general was lower in individuals with higher attachment avoidance and anxiety, even after statistically controlling for significant covariates such as current negative affect. Individuals in the clinical setting had higher levels of anxiety and avoidance as compared to non-clinical participants, and patients with a comorbid personality disorder (PD) had higher levels of both subscales of the ECR-RD in comparison to patients without a PD diagnosis. The ECR-RD was successfully used in a variety of research settings, ranging from social phobia [31], posttraumatic stress disorder [32] and revictimization [33], to mother–child-interventions [34], forensic psychiatry [35], medically unexplained pain conditions [36, 37], somatic symptom disorder [38], cardiovascular stress reaction [39], neuroscience [40], periodontal diseases [41], primary care [42], borderline personality disorder [43], bipolar disorder [44], childhood experiences [45], to research on spirituality [46].

Currently, there is a trend towards shortened versions of the ECR-R, mostly for assessment in large, community based or primary care samples. Notably, there are some brief measures adopted for research of attachment expectations to specific attachment figures [47], or for adolescents [48], and recently for adults in a sample of patients from primary care in Germany [49]. The latter 12-item version (ECR-RD12) was based on item analyses and principal components analysis (PCA) of a large sample of aggregated data from published and unpublished studies. The items were selected from the original 36 items of the ECR-RD according to their loadings on components derived from PCA. At the same time, this approach did not allow for a rigorous test of the assumed factor structure with regard to the underlying model of attachment-related anxiety and avoidance as core components of attachment [50]. In other words, we do not know whether the factor structure in adult German samples is in line with the theoretical two-dimensional model. This, however, is of special importance, as most published studies on the factor structure of the ECR-R from non-German samples indicated that effects of for example reverse coded items on these models need to be accounted for. At the same time, special approaches such partial disaggregation procedures that have been used for dealing with this topic in studies on the ECR-R do not provide very stringent tests of model fit [51–55].

### Current study

While the ECR-RD is a reliable and valid measure of adult attachment anxiety and avoidance, its length of 36 items still pose a limitation for its use in large-scale research and for screening^1^ purposes. Multicenter studies in psychiatry and related fields usually comprise a variety of instruments, thereby strongly limiting the number of possible items per construct. Large numbers of items reduce the compliance at the participant level, endangering recruitment and encouraging dropout. In addition, at the level of everyday practice in clinical settings, screening instruments need to be easy to use by the patients, and easy to interpret by the practitioner, which again calls for short screening versions of established measures. However, to our knowledge there is a lack of studies on a short version of the ECR-R in adults with a focus on testing the assumed factor structure by means of confirmatory factor analysis (CFA). Thus, the aim of the study was the evaluation and refinement of a short version of the ECR-RD attachment questionnaire (ECR-RD12) by means of CFA. We hypothesized that a) a short version will have acceptable psychometric properties, b), based on results from earlier studies (e.g., 29), that its scales will correlate with scores of another attachment instrument in a manner comparable to studies with the full version of the ECR-R, c) that living alone at the time of the study will be associated with higher levels of attachment anxiety and avoidance, and d) there is a negative association between attachment insecurity and general life satisfaction. Furthermore, we will e) provide representative norm-values of the resulting test scores for the German population as well as f) correlations between the full 36 item ECR-RD and the short form. For that, we conducted three studies, which are described in more detail below.

## Study 1

Aim of study 1 was to evaluate and refine an already existing brief version of the ECR-R (ECR-RD12), test differences in attachment scores between individuals currently living alone vs. in a relationship, explore relationships with age and gender, and provide norm values for the German population.

### Methods Study 1

#### Participants Study 1

Sample 1 was recruited by means of a nationally representative, face-to-face household survey conducted in 2013 by a specialized institute for demographic research (USUMA, Berlin), following a procedure specified by ADM Sampling Systems (F2F). The F2F allows for a random selection of individuals from a random selection of households from a random selection of area sample points based on a combination of geographical and statistical data that aims at representing the German general population through prior stratification procedures. The assessment was conducted in face-to-face interviews by trained, experienced interviewers after the participants gave their informed consent, and consisted of an interview- as well as a paper-and-pencil self-report part. In this sample, 4360 out of 4386 addresses were valid for the next step of the interview. Of those, 2526 interviews were conducted, with 2508 interviews eligible for analysis, resulting in a response-rate of 57.5%. Main reasons for not participating were refusal to participate of the household (13.6%) or the target person in the household (12.4%), no one of the household being home four times in a row (12.9%), and other reasons such as target person not being home (1.9%) or out of town (0.9%), or too ill to participate (0.4%). Of these 2508 participants, 36 participants (1.4%) did not fully complete the ECR-RD12, and further 44 participants (1.8%) did not complete the ECR-RD12 at all. These participants were excluded in our analyses, resulting in a total sample size of 2428 participants. The final sample consisted of 1291 female (53.2%) and 1137 male (46.8%) participants with an average age of 49.4 years (*SD* = 18.1). Regarding relationships, 1295 participants (53.3%) indicated that they were living together with their partner. For further descriptive data see Table 1.

**Table 1:** Demographic data in Study 1 (N = 2428)

	Total sample	
	*n*	%
	2428	100.0
*Sex*		
Male	1137	46.8
Female	1291	53.2
*Age groups*		
< 21	130	5.4
21–30	334	13.8
31–40	333	13.7
41–50	453	18.7
51–60	446	18.4
61–70	387	15.9
> 70	344	14.2
*Family status*		
Married	1158	47.7
Unmarried	683	28.1
Divorced	334	13.8
Widowed	253	10.4
Occupation		
Fulltime	979	40.3
Part-time	306	12.6
Unemployed	139	5.7
Retired	702	28.9
In training/further education	181	7.5
Other	19	.8
*Education*		
Missing	10	.4
Still in school	69	2.8
< 10 years	907	37.5
10 years	913	37.7
> 11 years	318	13.1
University/college degree	211	8.7

#### Measures Study 1

In Sample 1, a 12-item version (ECR-RD12) [49] of the German ECR-R [28] was used to assess attachment related anxiety and avoidance with regard to partner-related expectations and experiences. Each item is scored on a scale from one (strongly disagree) to seven (strongly agree), and mean values of the subscales are computed. For this version, four items of the avoidance scale are inverse coded. In addition, sociodemographic data was assessed, including relationship status.

#### Ethics statement Study 1

The study for Sample 1 was approved by the Institutional Review Board of the Medical Faculty of Leipzig University (050/13-ff).^2^

#### Statistical analyses Study 1

In Sample 1, we started with estimating a CFA based on the responses to the 12 items of the ECR-RD12. We specified two correlated latent factors representing attachment anxiety and avoidance. Cross-loadings and correlations between residuals were fixed to zero. To deal with the ordinal nature of the items, we used the polychoric correlation matrix and robust weighted least squares estimation [56]. To assess model fit, we inspected the comparative fit index (CFI; good fit: > 0.95), the Tucker-Lewis Index (TLI; good fit: > 0.95), the root mean square error of approximation (RMSEA; good fit: < 0.06), and the standardized root mean squared residual (SRMR; good fit: < 0.08; [57]). Internal consistency of the two scales was evaluated using McDonald’s Omega for ordinal items [58]. In case of insufficient fit indices, item reduction was based on modification indices of the CFA as well as content-related considerations. In addition, we report differences regarding gender and relationship status, and norm values for the German population. Norm values were computed based on the cumulative percentile distribution of scale scores, stratified for age groups and gender. Analyses were conducted using R version 4.0.3 [59] including the package lavaan (0.6–7; [60]) as well as IBM SPSS 25.

#### Results Study 1

Model fit of a two-dimensional CFA based on the 12 items of the ECR-RD12 was poor, χ^2^(53) = 8956.1, *p* < 0.001, CFI = 0.841, TLI = 0.802, RMSEA = 0.263, SRMR = 0.165. Thus, we reduced the item set by subsequently excluding the item involving the highest modification index, each time refitting the model and evaluating model fit. In this way we removed item 6 (due to a cross-loading on anxiety), item 7 (due to a cross-loading on anxiety), and item 1 (due to a cross-loading on avoidance and correlated residuals with item 2).^3^ To establish a measure with the same number of items for each scale, we also omitted item 10 (“I'm afraid that once a romantic partner gets to know me, he or she won't like who I really am.”). This decision was based on content, as the item represents the assumption of not being lovable (see cluster 27 from Fraley et al. [26]), as compared to the other items of the attachment anxiety subscale (see Table 2, Additional file 1: Table S5).

**Table 2:** Descriptive data for the ECR-RD8 in Study 1 (N = 2428)

	*Content*		*Md*	*M*	*SD*	Skewness	Kurtosis
ECR-RD8 Anxiety			2	2.39	1.35	.87	.04
ECRRD8_01	Ich mache mir oft Sorgen, dass mein Partner/meine Partnerin nicht bei mir bleiben will	I often worry that my partner will not want to stay with me	2	2.35	1.63	1.11	.24
ECRRD8_04	Ich befürchte, dass ich meinem Partner/meiner Partnerin weniger bedeute, als er/sie mir	I worry that romantic partners won’t care about me as much as I care about them	2	2.52	1.67	.91	− .22
ECRRD8_05	Ich habe den Eindruck, dass mein Partner/meine Partnerin nicht so viel Nähe möchte wie ich	I find that my partner(s) don't want to get as close as I would like	2	2.35	1.60	1.06	.14
ECRRD8_07	Es macht mich wütend, dass ich von meinem Partner/meiner Partnerin nicht die Zuneigung und Unterstützung bekomme, die ich brauche	It makes me mad that I don't get the affection and support I need from my partner	2	2.35	1.64	1.06	.11
ECR-RD8 Avoidance			2.5	2.89	1.70	.90	.04
ECRRD8_02 (i)	Ich fühle mich wohl damit, meine privaten Gedanken und Gefühle mit meinem Partner/meiner Partnerin zu teilen	I feel comfortable sharing my private thoughts and feelings with my partner	3	3.08	1.97	.72	− .63
ECRRD8_03 (i)	Es fällt mir leicht, mich auf meinen Partner/meine Partnerin zu verlassen	I find it easy to depend on romantic partners	2	2.94	1.96	.79	− .57
ECRRD8_06 (i)	Ich bespreche vieles mit meinem Partner/meiner Partnerin	I talk things over with my partner	2	2.81	1.94	.93	− .30
ECRRD8_08 (i)	Es fällt mir leicht, meinem Partner/meiner Partnerin gegenüber liebevoll zu sein	It's easy for me to be affectionate with my partner	2	2.72	1.86	1.00	− .01

*Md* = median; *M* = mean; *SD* = standard deviation; i = Reverse-scored items. ECR-RD8 = Experiences in Close Relationships-Revised Screening Version

**Fig. 1:**
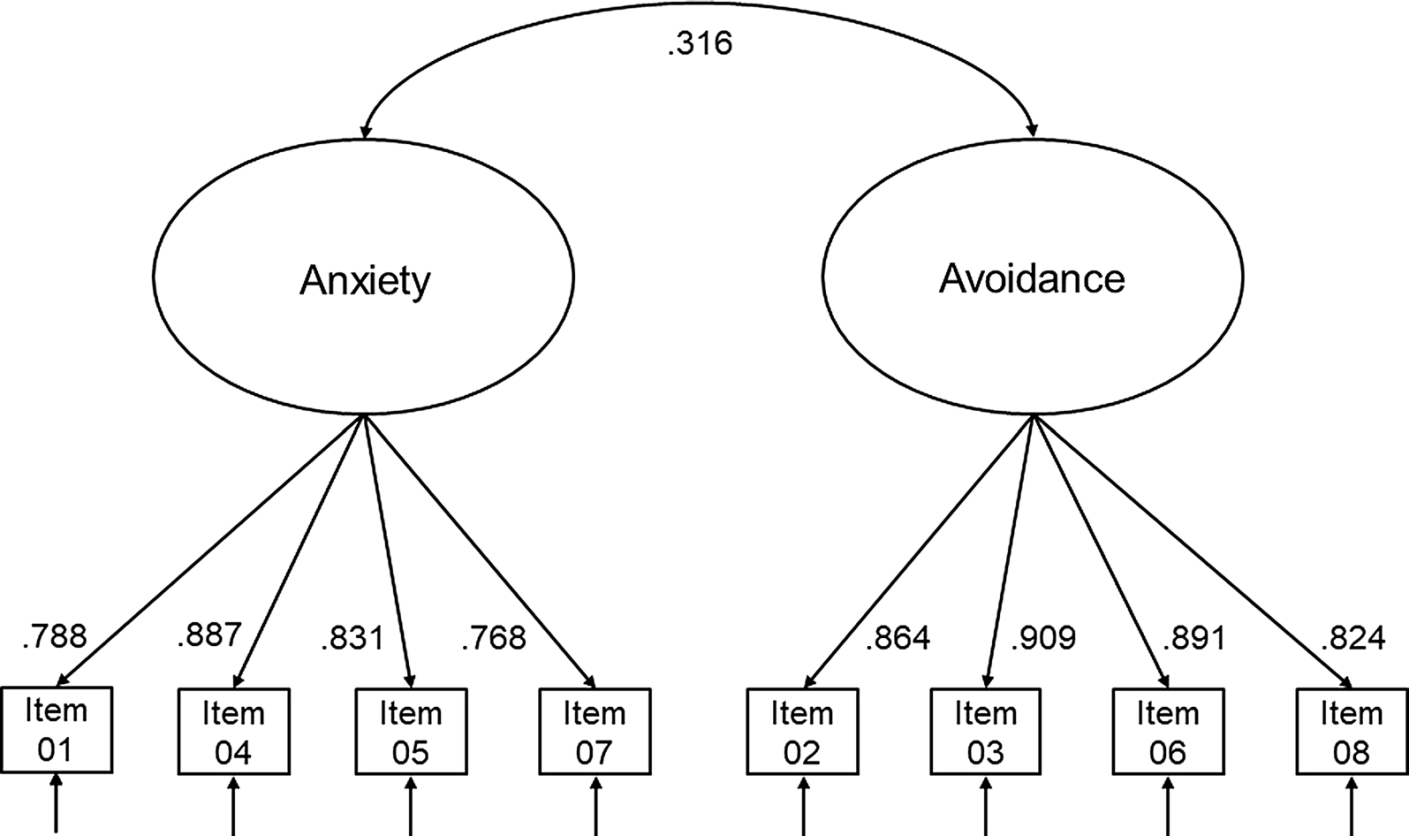
Factor loadings Study 1 (N = 2428). *Note* Anxiety = ECR-RD8 attachment-related anxiety; Avoidance = ECR-RD8 attachment-related avoidance. Numbers represent standardized estimates, residual variances are not displayed

The reduced 8-item version of the ECR-RD showed good model fit according to the majority of fit indices, χ^2^(19) = 438.1, *p* < 0.001, CFI = 0.989, TLI = 0.983, RMSEA = 0.095, SRMR = 0.044. Standardized factor loadings ranged from 0.77 to 0.91. Model-based internal consistency (McDonald’s Omega) was 0.87 for anxiety and 0.91 for avoidance. The correlation between the two latent factors was 0.32. Model parameters can be found in Fig. 1.

**Fig. 2:**
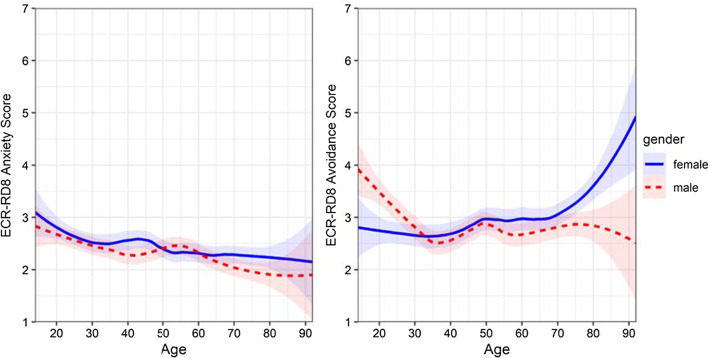
Non-linear associations between age and attachment styles for female and male participants in Study 1. *Note* ECR-RD8 = Experiences in Close Relationships-Revised Screening Version

We computed total (average) scores for the two 4-item scales. Female participants showed slightly higher values on anxiety compared to male participants, *t*(2426) = 2.11, *p* < 0.05, *d* = 0.09, but the sexes did not differ in terms of avoidance, *t*(2426) = 1.21, *p* = 0.23. Age was negatively associated with anxiety, *r* = − 0.13, *p* < 0.001, but not significantly associated with avoidance, *r* = 0.04, *p* = 0.07. However, when considering non-linear associations between age and attachment styles separately for female and male participants using local regression analyses, we found that young males and old females exhibited especially high scores of avoidance (see Fig. 2). Thus, we computed age- and gender-specific norm values for the two scales (see Additional file 1). Finally, we found that persons who currently live with a partner in the same household report lower values on anxiety, *t*(2426) = 7.91, *p* < 0.001, *d* = 0.32, and avoidance, *t*(2426) = 22.08, *p* < 0.001, *d* = 0.90, compared to persons who do not.

## Study 2

Aim of study 2 was to address correlations between the full version of the ECR-R and its resulting short form ECR-RD8.

### Methods Study 2

#### Participants and measures Study 2

The sample consisted of the dataset of the original German ECR-R evaluation. It comprised 1005 individuals from a non-clinical convenience sample, and 225 individuals in inpatient psychotherapy. The dataset is described in full detail elsewhere [28].

#### Ethics statement Study 2

Study 2 was positively evaluated by the Institutional Review Board of the Medical Faculty of the University of Göttingen (33/2/06).

#### Statistical analyses Study 2

To address the question of concordance between the ECR-RD8 and the ECR-RD, we re-analyzed the sample by calculating Pearson correlations between the ECR-RD8 subscales on the one hand, and the full ECR-R subscales and the ECR-RD subscales without the items used in the ECR-RD8 on the other hand. This provides a plausible range for the true statistical association between the full and the screening version. Analyses were conducted IBM SPSS 25.

#### Results Study 2

Pearson correlations between the ECR-RD8, the full ECR-RD, and the full ECR-RD subscales without the items of the ECR-RD8 were as follows: The 4-item ECR-RD8 anxiety subscale correlated with the 18-item full ECR-RD anxiety subscale at r = 0.92, and the reduced 14-item ECR-RD anxiety subscale at r = 0.85. The 4-item ECR-RD8 avoidance subscale correlated with the 18-item full ECR-RD avoidance subscale at r = 0.90, and the reduced 14-item ECR-RD avoidance subscale at r = 0.84.

## Study 3

Aim of our third study was to replicate the results concerning factor structure from study 1, and to assess relationships between the ECR-RD8 scale scores and another measure of attachment, relationship status and life satisfaction.

### Participants Study 3

Study 3 was recruited as an online convenience sample as part of another research project in German-speaking countries (Germany, Austria, and Switzerland). Individuals were invited through public social media platforms, and datasets were used if the ECR-RD8 was filled out without missing data. The sample consisted of 557 individuals, of which 447 identified as female (80.3%), 104 as male (18.7%), and six as “other” (1.1%). 248 (44.5%) reported an Austrian citizenship, 298 (51.9%) German citizenship, and 20 (3.6%) any other citizenship status. Regarding highest education, just four individuals (0.7%) had no formal graduation status form school, 33 (5.9%) had finished compulsory schooling, 221 (39.7%) high school. Seventy participants (12.6%) had finished vocational training, 62 (11.1%) a university of applied sciences degree, 69 (12.4%) a university BSc degree, 52 (9.3%) a university MSc degree, and 46 (8.3%) any other educational degree. In this sample, 319 individuals (57.3%) were currently in a relationship. General life satisfaction, measured on an ascending scale from one to ten, was *M* = 6.86 (*SD* = 1.93).

### Measures Study 3

In study 3, the ECR-RD8 was employed based on results from Sample 1. All four items of the avoidance subscale were inverse coded before mean values were computed. In addition to sociodemographic data and to allow for a test of convergent validity, the Relationship Questionnaire (RQ) [29, 30] was used as a brief measure of a general attachment-related self-concept. Individuals are presented with four paragraphs describing four different attachment styles according to the model of Bartholomew and Horowitz [29]: secure, dismissing, preoccupied, and fearful. They rate their agreement with each of these paragraphs on a seven-point scale from one (disagree strongly) to seven (agree strongly). For the RQ, ten individuals did not provide data, which result in a reduced sample size for correlational analyses. In addition, we assessed general life satisfaction on an ascending scale from one (low) to ten (high).

### Statistical analyses

In study 3, we applied CFA as described above to the 8 items, computed indices of internal consistency, correlations of the ECR-RD8 scale scores with the RQ to address questions of convergent validity, differences between individuals currently in a relationship vs. those currently not in a relationship, and associations with general life satisfaction. Analyses were conducted using R version 4.0.3 [59] as well as IBM SPSS 25.

### Results Study 3

In sample 3, the two-dimensional model of the ECR-RD8 showed acceptable model fit according to the majority of criteria, χ^2^(19) = 210.7, *p* < 0.001, CFI = 0.969, TLI = 0.940, RMSEA = 0.135, SRMR = 0.071. Internal consistency (McDonald’s Omega) was 0.83 for anxiety and 0.82 for avoidance. To address the question of convergent validity, we correlated ECR-RD8 subscales with RQ subscales. ECR-RD8 anxiety and avoidance subscales were weakly to moderately associated with each other. Both ECR-RD8 subscales correlated negatively with RQ security, and positively with RQ fearful. ECR-RD8 anxiety was slightly associated with RQ dismissing, and moderately with RQ preoccupied. ECR-RD8 avoidance was associated with RQ dismissing, but not with RQ preoccupied (see Table 3).

**Table 3:** Descriptive data for and zero-order correlations between ECR-RD8 and RQ subscales in Study 3 (N = 557)

	*M (SD)*	1	2	3	4	5	6
1 ECR-RD8 anxiety	3.06 (1.59)	–	.28**	− .33**	− .09*	.42**	.41**
2 ECR-RD8 avoidance	2.67 (1.34)		–	− .45**	.21**	.04	.46**
3 RQ secure	4.24 (1.82)			–	− .04	− .09*	− .53**
4 RQ dismissing	4.03 (1.87)				–	− .19**	.17**
5 RQ preoccupied	2.84 (1.80)					–	.18**
6 RQ fearful	3.85 (2.15)						–

*M* = mean; *SD* = standard deviation; ECR-RD8 = Experiences in Close Relationships-Revised Screening Version; RQ = Relationship Questionnaire. For the descriptive data on and correlations with the RQ, missing data resulted in a reduced sample size of N = 547

**p < .01; *p < .05

The ECR-RD8 anxiety subscale was significantly higher for individuals currently not in a relationship than for individuals in a relationship (M = 3.47 (SD = 1.56) vs. M = 2.76 (SD = 1.54), t(555) = − 5.42, *p* < 0.001, *d* = 0.46). The same was true for the avoidance subscale (M = 3.10 (SD = 1.37) vs. M = 2.35 (SD = 1.22), t(555) = − 6.75, *p* < 0.001, *d* = 0.58), indicating more attachment insecurity for currently single participants. More attachment anxiety (*r* = − 0.35, *p* < 0.01) and avoidance (*r* = − 0.39, *p* < 0.01) were correlated with lower levels of general life satisfaction.

## Discussion

In two independent samples, we found an eight-item version of the ECR-R to have largely acceptable fit indices in a two-dimensional CFA, and convergent validity with another attachment measure comparable to studies on the full ECR-R. In addition, individuals not living together with a partner (study 1) or without a current relationship (study 3) had significantly higher scores on attachment anxiety as well as avoidance. Those with higher levels of attachment anxiety and avoidance reported lower levels of life satisfaction, with moderate effect sizes. Statistical concordance between the scales of the screening version and the full scale were high (study 2). And finally, we present representative norm values from the German population for comparison in future research.

To our knowledge this is the first study to derive a short version of the ECR-R on the basis of CFA without making use of parceling-techniques. Because the original version by Fraley and colleagues [26] resulted from a procedure involving cluster-analytic techniques as a first step, followed by analyses based on item response theory, but the item reduction for the German 12-item version ECRR-D12 [49] was conducted via PCA, it was to be expected that an initial model-fit might be below the threshold usually expected.

The resulting items for attachment avoidance capture difficulties and/or discomfort with intimacy, i.e. the sharing of thoughts and feelings, relying on, communication with, and showing affection toward the partner. The resulting items for attachment anxiety address fear of abandonment, insecurity concerning the partner’s affection and support, and a wish for closeness. While those items of the ECR-RD8 do not capture all aspects of attachment insecurity as measured by the ECR-R full version, they nevertheless address a variety of attachment experiences and expectations. Results on validity are comparable to findings from other studies [28]. While we did not ask participants to fill out both the ECR-RD8 and the ECR-RD at the same time, the correlation between the scales of 0.85 to 0.92 for anxiety, and 0.84 to 0.90 for avoidance point towards a high statistical concordance between the instruments.

Strengths of the current study are the use of three independent samples, a solid statistical approach based on confirmatory factor analyses, and the development of norm values for the general population in Germany. Some limitations concern general aspects of attachment measurement. On the one hand, although most of the empirical results support an assessment on the two dimensions of attachment-related anxiety and avoidance [23], attachment questionnaires and interview-based measures show very little empirical overlap [26]. In other words, although an eight-item questionnaire captures some aspects of attachment insecurity, researchers should always be cautious to select an instrument which is best suited to their specific study question [22]. On the other hand, the question of whether the absence of attachment anxiety and avoidance is sufficient to define attachment security remains debated, especially when taking into account that the ECR-R measures attachment with regard to romantic relationships. This may also relate to the fact that all avoidance items are reverse-scored, indicating the absence of opening up and sharing relevant information and feelings with a partner. It remains unclear to what extent an influence of reverse scoring needs to be taken into account when interpreting the results, especially for the nonlinear effects of age by gender. Also, the sample that compared the ECR-RD8 to the RQ was not representative of the general population and consisted of about 80% participants identifying as female, restricting the generalizability of the results. However, the results are largely comparable to findings reported from the full 36-item version of the ECR-R [50]. Future studies should investigate more aspects of validity as well as temporal stability. Some indications on the use and further validity of the ECR-RD8 can be drawn from manuscripts that already successfully used the ECR-RD8, for example in psychotherapy trainees [61].

## Conclusion

The ECR-RD8 provides a useful and short measure to assess attachment anxiety and avoidance with good psychometric properties. The development of representative norm values will help to guide future research and interpretation of individual scores.

## Supplementary Information

**Additional file 1. Suppl. Table 1**. Age-specific T-values for ECR-RD8 avoidance scores in females.** Suppl. Table 2**. Age-specific T-values for ECR-RD8 anxiety scores in females.** Suppl. Table 3**. Age-specific T-values for ECR-RD8 avoidance scores in males.** Suppl. Table 4**. Age-specific T-values for ECR-RD8 anxiety scores in males.** Suppl. Table 5**. Frequency of response per item option in Study 1 (N = 2428).

## Data Availability

The datasets used and/or analyzed for the current study are available on reasonable request from the corresponding author.

## Abbreviation

ECR-R: Experiences in Close Relationships-Revised

## Footnotes

1 Technically one could argue that an ultra-brief version does not automatically qualify as a ‚screening ‘-instrument as we do not try to assess an outside criterion such as a specific mental disorder. However, we use this wording as we aim to capture the level of attachment related anxiety and avoidance as described above for contexts where a reduced number of items is needed.

2 All methods for all samples were carried out in accordance with relevant guidelines and regulations.

3 Fit indeces of the model changed in the process of removal of item 6 (χ^2^(43) = 5778.4, p < .001, CFI = .892, TLI = .862, RMSEA = .234, SRMR = .138), subsequent removal of item 7 (χ^2^(34) = 1757.6, p < .001, CFI = .966, TLI = .955, RMSEA = .145, SRMR = .079), and subsequent of removal of item 1 (χ^2^(26) = 491.8, p < .001, CFI = .989, TLI = .984, RMSEA = .086, SRMR = .042). The subsequent removal of item 10 resulted in the final reported model-fit.
