# Supplementary material for: Evaluation of a short version of the Experiences in Close Relationships-Revised questionnaire (ECR-RD8): results from a representative German sample
Source: BMC Psychol. 2021 Sep 14;9:140. doi: 10.1186/s40359-021-00637-z (PMC8439023; doi:10.1186/s40359-021-00637-z)
Supplement: Supplementary file 1 — Additional file 1. Suppl. Table 1. Age-specific T-values for ECR-RD8 avoidance scores in females. Suppl. Table 2. Age-specific T-values for ECR-RD8 anxiety scores in females. Suppl. Table 3. Age-specific T-values for ECR-RD8 avoidance scores in males. Suppl. Table 4. Age-specific T-values for ECR-RD8 anxiety scores in males. Suppl. Table 5. Frequency of response per item option in Study 1 (N = 2428). [file 40359_2021_637_MOESM1_ESM.docx]

Evaluation of a short version of the Experiences in Close Relationships – Revised questionnaire (ECR-RD8): results from a representative German sample

Johannes C. Ehrenthal*^1^, Johannes Zimmermann^2^, Katja Brenk-Franz^3^, Ulrike Dinger^4^, Henning Schauenburg^4^, Elmar Brähler^5,6^, Bernhard Strauß^3^

^1^Department of Psychology, University of Cologne, Cologne, Germany

^2^Department of Psychology, University of Kassel, Kassel, Germany

^3^Institute of Psychosocial Medicine, Psychotherapy and Psychooncology, Jena University Hospital, Jena, Germany

^4^Department of General Internal Medicine and Psychosomatics, University Hospital Heidelberg, Heidelberg, Germany

^5^Department of Medical Psychology and Medical Sociology, University Hospital Leipzig, Leipzig, Germany

^6^Department for Psychosomatic Medicine and Psychotherapy, University Medical Center Mainz, Mainz, Germany

*Corresponding author. Correspondence should be sent to Johannes C. Ehrenthal, Department of Psychology, University of Cologne, Bernhard-Feilchenfeld-Str. 11, 50969 Cologne, Germany. Email: [johannes.ehrenthal@uni-koeln.de](mailto:johannes.ehrenthal@uni-koeln.de).

Supplementary tables

Suppl. Table 1: Age-specific T-values for ECR-RD8 avoidance scores in females

|  | Age groups | | | | | | |
| --- | --- | --- | --- | --- | --- | --- | --- |
| Scores | <21  (n = 58) | 21-30  (n = 189) | 31-40  (n = 171) | 41-50  (n = 249) | 51-60  (n = 213) | 61-70  (n = 217) | >70  (n = 194) |
| 1 | 29 | 39 | 43 | 41 | 40 | 42 | 39 |
| 1.25 | 35 | 42 | 44 | 43 | 43 | 43 | 41 |
| 1.5 | 40 | 44 | 46 | 45 | 45 | 45 | 43 |
| 1.75 | 43 | 46 | 48 | 47 | 46 | 47 | 45 |
| 2 | 45 | 48 | 49 | 48 | 48 | 48 | 46 |
| 2.25 | 46 | 50 | 50 | 49 | 49 | 49 | 47 |
| 2.5 | 48 | 51 | 52 | 51 | 50 | 50 | 48 |
| 2.75 | 50 | 53 | 53 | 52 | 52 | 51 | 49 |
| 3 | 54 | 55 | 55 | 54 | 53 | 52 | 49 |
| 3.25 | 56 | 56 | 56 | 55 | 54 | 53 | 51 |
| 3.5 | 59 | 57 | 58 | 57 | 55 | 54 | 52 |
| 3.75 | 60 | 59 | 58 | 58 | 57 | 55 | 53 |
| 4 | 63 | 60 | 60 | 59 | 58 | 57 | 55 |
| 4.25 | 64 | 62 | 61 | 60 | 59 | 58 | 56 |
| 4.5 | 64 | 63 | 62 | 60 | 60 | 58 | 57 |
| 4.75 | 64 | 65 | 64 | 62 | 60 | 59 | 58 |
| 5 | 64 | 66 | 64 | 62 | 61 | 59 | 59 |
| 5.25 | 66 | 66 | 65 | 64 | 61 | 60 | 59 |
| 5.5 | 66 | 67 | 65 | 64 | 63 | 61 | 60 |
| 5.75 | 68 | 67 | 66 | 65 | 63 | 62 | 60 |
| 6 | 68 | 68 | 67 | 65 | 64 | 62 | 61 |
| 6.25 | 68 | 68 | 67 | 65 | 64 | 63 | 61 |
| 6.5 | 68 | 68 | 68 | 66 | 64 | 63 | 61 |
| 6.75 | 68 | 69 | 68 | 67 | 64 | 64 | 62 |
| 7 | >68 | >69 | >68 | >67 | >64 | >64 | >62 |

Note. Norm values based on the cumulative percentile distribution of scale scores.

Suppl. Table 2: Age-specific T-values for ECR-RD8 anxiety scores in females

|  | Age groups | | | | | | |
| --- | --- | --- | --- | --- | --- | --- | --- |
| Scores | <21  (n = 72) | 21-30  (n = 145) | 31-40  (n = 162) | 41-50  (n = 204) | 51-60  (n = 233) | 61-70  (n = 170) | >70  (n = 151) |
| 1 | 39 | 39 | 42 | 43 | 44 | 45 | 45 |
| 1.25 | 42 | 44 | 44 | 45 | 46 | 47 | 46 |
| 1.5 | 44 | 46 | 46 | 46 | 48 | 49 | 48 |
| 1.75 | 46 | 48 | 48 | 48 | 50 | 51 | 49 |
| 2 | 47 | 49 | 49 | 49 | 51 | 51 | 51 |
| 2.25 | 49 | 50 | 50 | 51 | 53 | 52 | 52 |
| 2.5 | 50 | 52 | 52 | 52 | 54 | 54 | 53 |
| 2.75 | 52 | 53 | 54 | 53 | 55 | 56 | 55 |
| 3 | 53 | 54 | 55 | 54 | 56 | 57 | 57 |
| 3.25 | 54 | 56 | 56 | 56 | 57 | 58 | 58 |
| 3.5 | 55 | 57 | 58 | 57 | 59 | 59 | 60 |
| 3.75 | 55 | 57 | 60 | 59 | 60 | 61 | 62 |
| 4 | 56 | 58 | 62 | 60 | 61 | 63 | 64 |
| 4.25 | 58 | 59 | 64 | 62 | 63 | 64 | 64 |
| 4.5 | 59 | 61 | 68 | 63 | 65 | 65 | 66 |
| 4.75 | 59 | 62 | 71 | 64 | 66 | 66 | 70 |
| 5 | 61 | 65 | 75 | 66 | 70 | 66 | 70 |
| 5.25 | 64 | 66 | >75 | 66 | 70 | 67 | 70 |
| 5.5 | 64 | 69 | >75 | 68 | 70 | 68 | 70 |
| 5.75 | 66 | 70 | >75 | 70 | 72 | 70 | 70 |
| 6 | 66 | 71 | >75 | 71 | 72 | 71 | 73 |
| 6.25 | 71 | 76 | >75 | 73 | 72 | 76 | 76 |
| 6.5 | >71 | >76 | >75 | 77 | 72 | 76 | 76 |
| 6.75 | >71 | >76 | >75 | >77 | 73 | >76 | 76 |
| 7 | >71 | >76 | >75 | >77 | >73 | >76 | >76 |

Note. Norm values based on the cumulative percentile distribution of scale scores.

Suppl. Table 3: Age-specific T-values for ECR-RD8 avoidance scores in males

|  | Age groups | | | | | | |
| --- | --- | --- | --- | --- | --- | --- | --- |
| Scores | <21  (n = 72) | 21-30  (n = 145) | 31-40  (n = 162) | 41-50  (n = 204) | 51-60  (n = 218) | 61-70  (n = 185) | >70  (n = 151) |
| 1 | 28 | 37 | 41 | 41 | 42 | 41 | 43 |
| 1.25 | 31 | 38 | 44 | 43 | 44 | 43 | 44 |
| 1.5 | 36 | 40 | 45 | 45 | 46 | 46 | 45 |
| 1.75 | 38 | 41 | 47 | 47 | 47 | 48 | 47 |
| 2 | 38 | 44 | 50 | 49 | 49 | 49 | 48 |
| 2.25 | 41 | 45 | 51 | 50 | 50 | 51 | 50 |
| 2.5 | 46 | 48 | 52 | 52 | 52 | 52 | 52 |
| 2.75 | 47 | 50 | 53 | 52 | 53 | 53 | 53 |
| 3 | 48 | 51 | 54 | 54 | 55 | 54 | 55 |
| 3.25 | 50 | 52 | 56 | 55 | 55 | 55 | 55 |
| 3.5 | 52 | 55 | 57 | 56 | 57 | 57 | 57 |
| 3.75 | 53 | 56 | 59 | 57 | 58 | 57 | 57 |
| 4 | 55 | 58 | 61 | 59 | 60 | 59 | 59 |
| 4.25 | 57 | 59 | 62 | 60 | 60 | 60 | 59 |
| 4.5 | 57 | 61 | 63 | 61 | 61 | 60 | 60 |
| 4.75 | 60 | 62 | 63 | 62 | 61 | 61 | 61 |
| 5 | 60 | 62 | 64 | 63 | 62 | 61 | 61 |
| 5.25 | 61 | 63 | 65 | 63 | 63 | 61 | 62 |
| 5.5 | 62 | 64 | 67 | 66 | 64 | 63 | 62 |
| 5.75 | 62 | 64 | 67 | 68 | 65 | 63 | 63 |
| 6 | 63 | 67 | 67 | 68 | 66 | 64 | 63 |
| 6.25 | 63 | 67 | 67 | 69 | 69 | 64 | 64 |
| 6.5 | 63 | 67 | 67 | 70 | 69 | 65 | 65 |
| 6.75 | 63 | 67 | 67 | 70 | 70 | 65 | 65 |
| 7 | >63 | >67 | >67 | >70 | >70 | >65 | >65 |

Note. Norm values based on the cumulative percentile distribution of scale scores.

Suppl. Table 4: Age-specific T-values for ECR-RD8 anxiety scores in males

|  | Age groups | | | | | | |
| --- | --- | --- | --- | --- | --- | --- | --- |
| Scores | <21  (n = 72) | 21-30  (n = 145) | 31-40  (n = 162) | 41-50  (n = 204) | 51-60  (n = 218) | 61-70  (n = 185) | >70  (n = 151) |
| 1 | 41 | 40 | 43 | 44 | 42 | 46 | 47 |
| 1.25 | 42 | 43 | 45 | 46 | 44 | 48 | 48 |
| 1.5 | 43 | 45 | 47 | 48 | 46 | 49 | 49 |
| 1.75 | 45 | 47 | 48 | 50 | 48 | 51 | 52 |
| 2 | 46 | 49 | 50 | 51 | 50 | 52 | 53 |
| 2.25 | 48 | 51 | 51 | 52 | 52 | 54 | 56 |
| 2.5 | 50 | 52 | 53 | 54 | 54 | 55 | 58 |
| 2.75 | 51 | 53 | 54 | 55 | 55 | 57 | 59 |
| 3 | 53 | 54 | 55 | 56 | 56 | 57 | 60 |
| 3.25 | 55 | 57 | 56 | 57 | 58 | 58 | 61 |
| 3.5 | 56 | 59 | 58 | 59 | 59 | 59 | 62 |
| 3.75 | 57 | 60 | 59 | 61 | 61 | 61 | 63 |
| 4 | 60 | 62 | 60 | 64 | 63 | 63 | 66 |
| 4.25 | 62 | 64 | 62 | 64 | 64 | 65 | 67 |
| 4.5 | 63 | 66 | 64 | 65 | 65 | 67 | 68 |
| 4.75 | 65 | 68 | 69 | 67 | 67 | 68 | 71 |
| 5 | 66 | 70 | 71 | 68 | 68 | 70 | 72 |
| 5.25 | 66 | 75 | 71 | 72 | 69 | 70 | 72 |
| 5.5 | 67 | 75 | 72 | 73 | 70 | 70 | 72 |
| 5.75 | 67 | 75 | 75 | 76 | 70 | 75 | 72 |
| 6 | 67 | >75 | >75 | >76 | 70 | 75 | 75 |
| 6.25 | 69 | >75 | >75 | >76 | 71 | 75 | 75 |
| 6.5 | 72 | >75 | >75 | >76 | 74 | >75 | 75 |
| 6.75 | 72 | >75 | >75 | >76 | 74 | >75 | 75 |
| 7 | >72 | >75 | >75 | >76 | >74 | >75 | >75 |

Note. Norm values based on the cumulative percentile distribution of scale scores.

Suppl. Table 5. Frequency of response per item option in Study 1 (N = 2428).

|  |  | *Frequency (%) of response per item option* | | | | | | |
| --- | --- | --- | --- | --- | --- | --- | --- | --- |
| ECR-RD8 Anxiety |  | 1 | 2 | 3 | 4 | 5 | 6 | 7 |
| ECRRD8_01 | I often worry that my partner will not want to stay with me. | 45.3 | 19.6 | 12.0 | 10.0 | 7.1 | 4.0 | 2.1 |
| ECRRD8_04 | I worry that romantic partners won’t care about me as much as I care about them. | 39.5 | 20.4 | 12.9 | 12.1 | 7.7 | 5.2 | 2.0 |
| ECRRD8_05 | I find that my partner(s) don't want to get as close as I would like. | 44.8 | 19.4 | 12.2 | 11.0 | 7.1 | 3.8 | 1.7 |
| ECRRD8_07 | It makes me mad that I don't get the affection and support I need from my partner. | 46.6 | 17.2 | 11.9 | 11.3 | 6.8 | 4.2 | 2.0 |
| ECR-RD8 Avoidance |  |  | | | | | | |
| ECRRD8_02 (i) | I feel comfortable sharing my private thoughts and feelings with my partner. | 28.1 | 20.0 | 16.1 | 13.8 | 6.5 | 4.7 | 10.9 |
| ECRRD8_03 (i) | I find it easy to depend on romantic partners. | 32.1 | 20.5 | 13.9 | 11.7 | 7.5 | 4.9 | 9.4 |
| ECRRD8_06 (i) | I talk things over with my partner. | 35.0 | 21.0 | 14.2 | 10.5 | 5.9 | 4.0 | 9.3 |
| ECRRD8_08 (i) | It's easy for me to be affectionate with my partner. | 35.9 | 20.3 | 15.4 | 12.1 | 5.4 | 2.9 | 8.9 |

Note. *Md* = Median; *M* = Mean; *SD* = Standard Deviation; i = Reverse-scored items. ECR-RD8 = Experiences in Close Relationships – Revised Screening Version. Response option 1 is labeled “strongly disagree”, response option 7 is labeled “strongly agree”.
